# Resource Concentration and Clustering in Replicator Dynamics with Stochastic Reset Events

**DOI:** 10.3390/e25010099

**Published:** 2023-01-03

**Authors:** Ignacio T. Gómez Garay, Damián H. Zanette

**Affiliations:** 1 Centro Atómico Bariloche and Instituto Balseiro, Comisión Nacional de Energía Atómica, Universidad Nacional de Cuyo, Av. E. Bustillo 9500, San Carlos de Bariloche 8400, Argentina; 2 Consejo Nacional de Investigaciones Científicas y Técnicas, Bariloche 8400, Argentina

**Keywords:** replicator population, stochastic resetting, resource distribution, anomalous fluctuations, clustering

## Abstract

As a model for economic and ecological systems, replicator dynamics represent a basic form of agent competition for finite resources. Here, we investigate the effects of stochastic resetting in this kind of processes. Random reset events abruptly lead individual resources to a small value from which dynamics must start anew. Numerical results show that resource distribution over the population of competing agents develops highly nonuniform profiles, exhibiting clustering and fluctuations with anomalous dependence on the population size. This non-standard statistical behavior jeopardizes an analytical treatment based on mean-field assumptions. We propose alternative simplified analytical approaches which provide a stylized description of entropy evolution for the clustered distribution of resources and explain the unusually slow decrease of fluctuations.

## 1. Introduction

In theoretical biology, a *replicator* is an abstract unit capable of creating copies of itself through interaction with the environment [1,2]. This very generic concept—which provides a unified tool for studying evolutionary dynamics at several levels—encompasses such entities as nucleic-acid molecules (RNA and DNA), genes, cells, and, of course, living organisms. In the theory of cultural evolution, an analogous notion applies to memes, the units of cultural information, thus extending the same theoretical framework to social and economic phenomena [3]. The concept of replicator turned out to be especially fruitful within evolutionary game theory, as a model for biological evolution under natural selection. In this context, replicators represent strategies whose individual profit, measured by their relative reproduction success, depends on both their intrinsic fitness and their mutual interaction [4].

Replicator dynamics is a mathematical model, used in evolutionary game theory, that describes how the relative prevalence of different strategies changes in time [5,6]. If, in a large population, $x_{i}\left(t\right)$ is the fraction of players adopting strategy *i* at time *t*, replicator dynamics prescribe that

(1) $${\dot{x}}_{i}=x_{i}\left[f_{i}\left(x\right)-\sum_{j=1}^{N}f_{j}\left(x\right)x_{j}\right],$$

(i=1,2,⋯,N), where $f_{i}\left(x\right)$ denotes the fitness of strategy *i*, and generally depends on all the components of $x=(x_{1},x_{2},\cdots ,x_{N})$. It can be seen that the *N*-dimensional simplex, given by $\sum_{i}x_{i}=1$ with $x_{i}\geq 0$ for all *i*, is invariant under Equation (1), and also acts as a global attractor for all non-negative initial conditions. From the perspective of population dynamics, Equation (1) can be interpreted as the time evolution of *N* interacting species with fitnesses $f_{i}\left(x\right)$, additionally subjected to a global mechanism of growth limitation, given by the second term in the brackets, which asymptotically constrains populations to the subspace where $\sum_{i}x_{i}=1$. In this work, we adopt a similar interpretation, where $x_{i}$ represents the resources (richness) of an economic agent *i* in a population of *N* interacting agents.

In the simplest version of replicator dynamics, all fitnesses are constant: $f_{i}\left(x\right)=\lambda_{i}$ for all *i* [7]. In this situation, the first term in the right-hand side of Equation (1) induces an exponential growth of the resources $x_{i}$, at rate $\lambda_{i}$. The opposing effect of the second term, however, limits this growth. For sufficiently long times, in fact, the system approaches the *N*-dimensional simplex. The outcome of these contrary trends is that, asymptotically, the replicator with maximal fitness accumulates all the resources. Namely, for t→∞,

(2) $$x_{i}=\left\{\begin{array}{cc} 1 & \mathrm{if}\;\lambda_{i}=\max\left\{\lambda_{1},\lambda_{2},\cdots ,\lambda_{N}\right\},\\ 0 & \mathrm{otherwise}. \end{array}\right.$$

Thus, with constant fitnesses, the population always ends in a state where resources are trivially concentrated in just one agent. If two or more agents have identical maximal fitnesses, all the resources become shared between them in proportions depending on the initial values $x_{i}\left(0\right)$.

Our aim in this paper is to study the effect of reset events on the replicator dynamics with constant fitnesses. Resetting is a stochastic mechanism by which a dynamical variable—in the present case, $x_{i}\left(t\right)$—is occasionally brought to a prefixed value, from which its dynamics start anew. This mechanism is able to severely modify the statistical behavior of a dynamical system [8]. In the present case, we expect it to inhibit the accumulation of resources by a single agent or a small group of agents, bringing about a nontrivial resource distribution over the replicator population. To gain insight into the overall behavior of our model, which we present in Section 2, Section 3 is devoted to the numerical and analytical study of the case of a single replicator. In Section 4, we show that the combined effect of replicator dynamics and resetting in a large population with identical fitnesses results in anomalous statistical properties, with an extremely slow decrease of fluctuations as the population size grows. This unusual feature is accompanied by clustering in the amount of individual resources, which, over time, sustains a highly heterogeneous resource distribution over the population. Analytical arguments based on a toy two-cluster model are proposed to explain these numerical observations. Finally, Section 5 is devoted to discussing our main results.

## 2. Replicators with Resetting

Stochastic resetting was initially introduced as a mechanism of unbounded growth limitation in the context of demographic dynamics [9,10]. Remarkably, when combined with multiplicative (exponential) growth, it gives rise to long-time power-law distributions for the relevant variables [10,11]. It can therefore be used as a model for the emergence of such distributions in the broad class of phenomena where they are observed [12], ranging from biological taxon abundances [13] to economic resource sharing [14]. Since its introduction more than two decades ago, the statistical effects of stochastic resetting have been studied in a wide variety of dynamical processes, such as transport on networks [15], hydrologic phenomena [16], RNA kinetics [17], and active-particle motion [18], among many others [8].

As described in the Introduction, stochastic resetting acts on a variable x(t), whose evolution is otherwise governed by certain dynamical rules, instantaneously bringing its value to a prefixed level *u*. Reset events are distributed at random along time, and the evolution of x(t) begins de novo after each resetting. Such events emulate the effect of sudden crises or catastrophic occurrences, where the state of the system under study suffers an abrupt change in a short time [19]. This kind of phenomenon is not uncommon in social and economic contexts [11,20,21].

In replicator dynamics with constant fitnesses $\lambda_{i}$, we introduce reset events by proposing

(3) $${\dot{x}}_{i}=x_{i}\left(\lambda_{i}-\sum_{j=1}^{N}\lambda_{j}x_{j}\right)+\left(u_{i}-x_{i}\right)P_{i}\left(t\right),$$

(i=1,2,⋯,N; cf. Equation (1)). Here,

(4) $$P_{i}\left(t\right)=\sum_{k}\delta \left(t-t_{i,k}\right)$$

represents a Poisson (or shot [22]) noise signal, δ(t) being the Dirac delta function. For each *i*, the reset times $t_{i,k}$ (k=1,2,⋯) are randomly distributed with uniform frequency $q_{i}$, so that the average lapse between $t_{i,k}$ and $t_{i,k+1}$ is $q_{i}^{-1}$ for all *k*. The prefactor $u_{i}-x_{i}$ in the last term of Equation (3) insures that each reset event brings $x_{i}\left(t\right)$ to the reset value $u_{i}$. The Markovian stochastic Equation (3) can be dealt with by means of a series of standard methods, notably, the Chapman-Kolmogorov equation, which governs the joint probability distribution of the resources $x_{i}\left(t\right)$ [22]. It can also be treated numerically, by a rather intuitive implementation of the Poisson process along discretized time [23]. In the following sections, we use these techniques to study the collective dynamics of the replicator population with resetting.

## 3. Dynamics of a Single Replicator with Resetting

As a first step in the analysis of our model, it is instructive to study the case of a single replicator, N=1. Equation (3) becomes

(5) $$\dot{x}=\lambda x\left(1-x\right)+\left(u-x\right)P\left(t\right),$$

with $P\left(t\right)=\sum_{k}\delta \left(t-t_{k}\right)$. The random reset times $t_{k}$ have frequency *q*. The first term in the right-hand side of Equation (5) makes it clear that, for a single replicator, the deterministic contribution to the dynamics is equivalent to logistic growth [24]. Due to arbitrariness in the choice of time units, the system has two independent parameters only: the ratio q/λ, and the reset value *u*.

**Figure 1:**
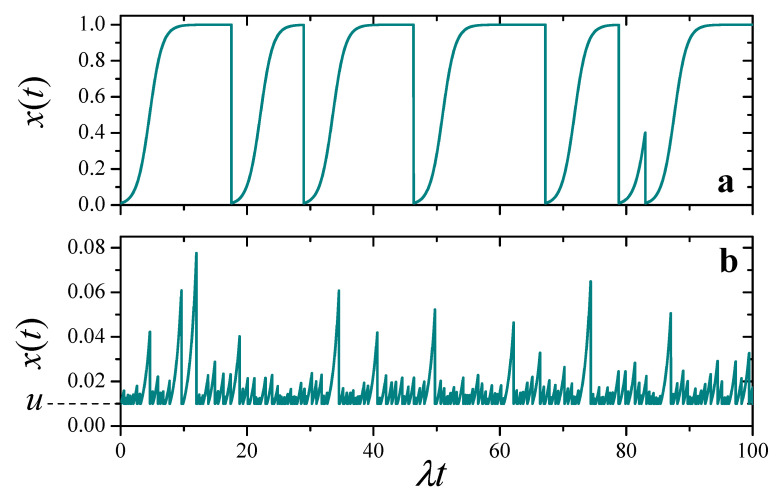
Two realizations of the solution to the stochastic Equation (5), for u=0.01 and different values of the ratio q/λ. (**a**) q/λ=0.1. (**b**) q/λ=2.5. Note different scales in the vertical axes.

Figure 1 shows a pair of realizations of x(t), for u=0.01 and two values of q/λ, exhibiting qualitatively different behavior. For a relatively small resetting frequency, q/λ=0.1 (upper panel), x(t) usually has enough time to reach the zone of logistic saturation, just below the level of maximal resources (x=1). The evolution is only occasionally punctuated by reset events to x=u. On the other hand, when the resetting frequency is larger (q/λ=2.5, lower panel), x(t) barely transits the zone of exponential growth before it is interrupted by a reset event. In this latter situation, the evolution is very similar to the case where the deterministic part of the dynamics is purely multiplicative, which we have analyzed in detail in a recent contribution [19].

Assuming that the stochastic process represented by Equation (5) reaches a stationary regime for long times, the stationary distribution for *x*, $f^{\mathrm{st}}\left(x\right)$, can be obtained from the Chapman-Kolmogorov equation

(6) $$\frac{\partial }{\partial t}f\left(x,t\right)+\frac{\partial }{\partial x}\left[v(x)f(x,t)\right]=q\delta \left(u-x\right)-qf\left(x,t\right)$$

by fixing $\partial_{t}f\equiv 0$. In the left-hand side of this equation, the second term represents the probability drift induced by the deterministic logistic dynamics, with v(x)=λx(1−x). The two terms in the right-hand side are gain and loss contributions originating in reset events. The positive gain term is different from zero only at the reset value x=u, while the negative term represents uniform probability loss at frequency *q* for all *x*. On the whole, of course, the two terms compensate each other. For x≠u, the effect of the delta-like gain term can interpreted as a boundary condition which connects the solution in the intervals x<u and x>u through the relation $v\left(u^{+}\right)f\left(u^{+},t\right)-v\left(u^{-}\right)f\left(u^{-},t\right)=q$ for t>0, as obtained from integration of Equation (6) around x=u. Using this boundary condition, the stationary solution reads

(7) $$f^{\mathrm{st}}\left(x\right)=\frac{q}{\lambda }{\left(\frac{1-u}{u}\right)}^{-q/\lambda }x^{-1-q/\lambda }{\left(1-x\right)}^{-1+q/\lambda }$$

for u≤x<1, and $f^{\mathrm{st}}\left(x\right)=0$ otherwise. This time-independent distribution behaves as a power law both for small and large values of *x*. For q/λ>1, the exponent of 1−x is positive, and the distribution has a maximum at x=u while it decays to zero as x→1.

**Figure 2:**
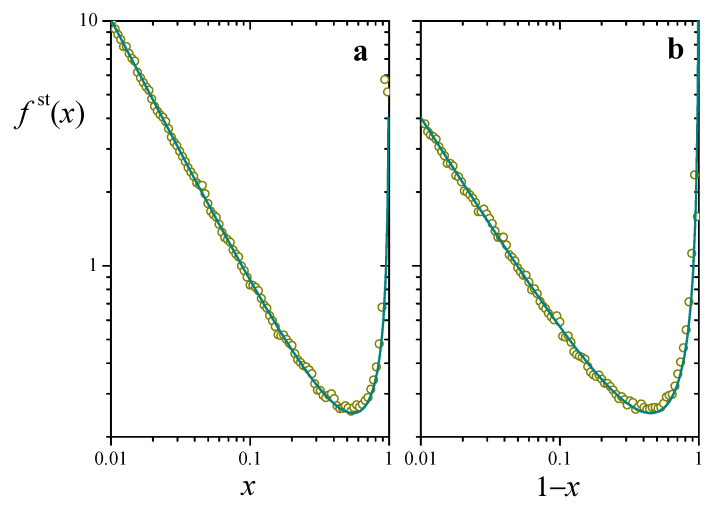
Stationary probability distribution for the solution to the stochastic Equation (5), $f^{\mathrm{st}}\left(x\right)$, (**a**) as a function of *x* and (**b**) as a function of 1−x, for u=0.01 and q/λ=0.1. The line stands for the analytical expression (7). Symbols correspond to a 100-column histogram, built from $4\times 10^{5}$ samples of x(t) taken from a numerical realization of Equation (5) every 10 time units. The numerical solution was realized by means of a standard finite-difference algorithm with a time step of $10^{-3}$ time units.

For q/λ<1, on the other hand, $f^{\mathrm{st}}\left(x\right)$ exhibits a bimodal profile, with a local maximum at x=u and a divergence at x=1. This case is illustrated in Figure 2, where we plot the distribution as a function of both *x* (left panel) and 1−x (right panel) for u=0.01 and q/λ=0.1. The log-log axes emphasize the power-law dependence toward the two ends. Excellent agreement between analytical and numerical results supports the assumption of a well-defined long-time stationary regime for the stochastic process. The bimodal concentration of resources at the extreme values, with the ensuing depletion in the intermediate zone, is a direct consequence of the competing effect of logistic growth, which favors accumulation near the maximum, and of reset events, which populate the zone of lower resources.

## 4. Fluctuations and Clustering in Large Homogeneous Populations

Turning now the attention to the case with N>1, we consider homogeneous replicator populations, in which the parameters $u_{i}$, $\lambda_{i}$, and $q_{i}$ in Equation (3) are the same for all agents. In this situation, agents differ from each other in the individual realizations of the sequence of stochastic reset events only. This homogeneity implies that none of them has an *a priori* advantage based on fitness, or on the frequency and strength of resetting. Thus, any nontrivial emergent collective behavior should be ascribed to the randomness in the time distribution of reset events.

For a homogeneous population, Equation (3) reads

(8) $${\dot{x}}_{i}=\lambda x_{i}\left(1-x_{T}\right)+\left(u-x_{i}\right)P_{i}\left(t\right),$$

with $P_{i}\left(t\right)$ given as in Equation (4) with the same resetting frequency *q* for all *i*. In turn,

(9) $$x_{T}=\sum_{j=1}^{N}x_{j}$$

stands for the total resources over the population. Assuming that, as in the case of N=1, the system attains a well-defined stationary state for long times, we expect that $x_{T}$ reaches a constant value if *N* is large enough. Of course, this requires that resource fluctuations are self-averaging over time and over the ensemble. If these conditions are fulfilled, the stationary distribution for individual resources satisfies Equation (6) with, now, $v\left(x\right)=\lambda x\left(1-x_{T}\right)$. The solution is

(10) $$f^{\mathrm{st}}\left(x\right)=\frac{qu^{q/\lambda (1-x_{T})}}{\lambda (1-x_{T})}x^{-1-q/\lambda (1-x_{T})},$$

for u≤x<1 and 0 otherwise. The absence of a logistic nonlinearity in Equation (8) determines that $f^{\mathrm{st}}\left(x\right)$ is now a pure power law; cf. (7).

The value of $x_{T}$ in Equation (10) must be obtained self-consistently, requiring that it coincides with the total resources calculated from the distribution $f^{\mathrm{st}}\left(x\right)$, namely

(11) $$x_{T}=N\int_{u}^{1}xf^{\mathrm{st}}\left(x\right)dx=\frac{Nqu}{q-\lambda (1-x_{T})}.$$

The only positive solution to this self-consistency equation is

(12) $$x_{T}=\frac{\lambda /q-1+\sqrt{{\left(\lambda /q-1\right)}^{2}+4Nu\lambda /q}}{2\lambda /q}.$$

For a given value of Nu, the total resources vary monotonically from $x_{T}\approx 1-q/\lambda \approx 1$ for q≪λ to $x_{T}\approx Nu$ for q≫λ. In the first limit, when the resetting frequency is negligible, the population is driven by almost purely replicator dynamics, and one single agent typically concentrates all the resources. When, on the other hand, reset events are dominant, the *N* agents always have resources close to the minimal value *u*. The corresponding distributions are

(13) $$f^{\mathrm{st}}\left(x\right)\approx \left\{\begin{array}{cc} {\left(u^{-1}-1\right)}^{-1}x^{-2} & \mathrm{for}\;q\ll \lambda ,\\ {\left(u^{-q/\lambda }-1\right)}^{-1}x^{-1-q/\lambda } & \mathrm{for}\;q\gg \lambda . \end{array}\right.$$

In the remaining of this paper, we fix the attention on the case q<λ. Indeed, much as in the case of N=1 analyzed in Section 3, for q>λ—when reset events dominate over resource growth—the replicator dynamics hardly manifests itself, and evolution does not essentially differ from that of a system of non-interacting multiplicative elements with resetting ([19], cf. Figure 1b). For brevity, numerical results are presented for just a few parameter sets, which we have found to be representative of more general situations.

**Figure 3:**
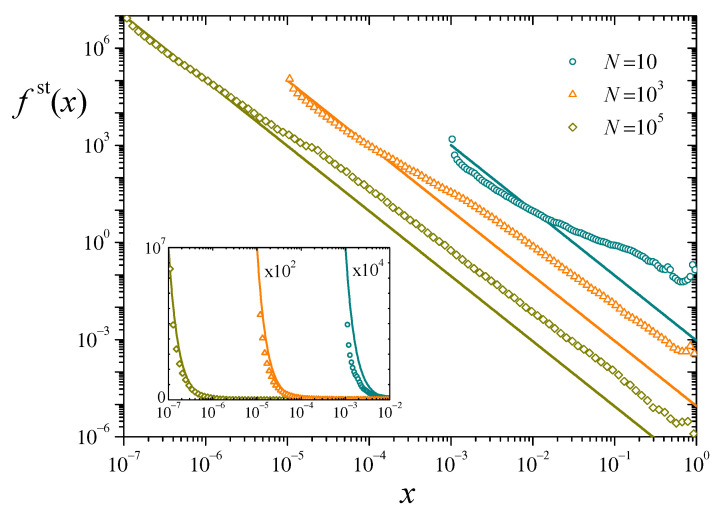
Symbols: Numerical estimation of the stationary distribution $f^{\mathrm{st}}\left(x\right)$ for three values of *N*, with Nu=0.01 and q/λ=0.1. Lines: Analytical solution (10) to the stationary Chapman-Kolmogorov equation, for the same parameters. Inset: The same data in log-linear scales, for a better appraisal in the upper part of the vertical axis. The data for N=10 and $10^{3}$ have been scaled by the factors indicated in the plot.

Following the same numerical techniques used in the case of a single replicator, we have computed the stationary distribution of individual resources for populations of different sizes, with Nu=0.01 and q/λ=0.1. According to the analytical result of Equation (12), all these systems have the same total resources, $x_{T}\approx 0.901$. Symbols in Figure 3 show histograms of $f^{\mathrm{st}}\left(x\right)$ for three values of *N*, analogous to those presented in Figure 2 for N=1. Lines stand for the corresponding analytical prediction (10).

It is apparent that, although numerical and analytical results follow the same general trend in the distribution of resources, there are important systematic deviations along the whole interval of the variable *x*. The deviations decrease in magnitude as the population grows, but are still non-negligible for a large system of $10^{5}$ replicators. For this size and large *x*, the slopes of the power-law tails in the numerical estimation and the analytical prediction are very similar but, as for the values of the distributions, the former are about one order of magnitude above the latter. The difference has the opposite sign at small *x*, as shown in the inset. We show in the following paragraphs that these discrepancies originate in the anomalous statistical behavior of the total resources $x_{T}\left(t\right)$. Its fluctuations along time, in fact, decay very slowly with the system size *N*. This indicates that our assumption that $x_{T}$ is constant, used to solve the stationary Chapman-Kolmogorov equation, may only hold for extremely large populations, drastically limiting the usefulness of the analytical approach in this kind of systems.

### 4.1. Anomalous Fluctuations of Total Resources

**Figure 4:**
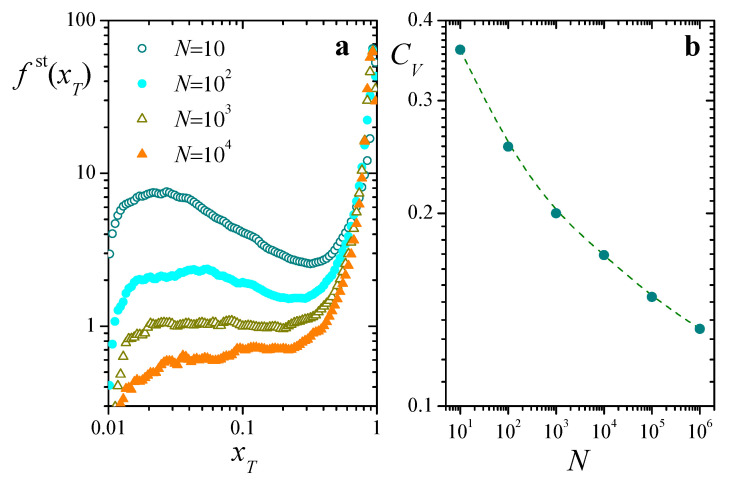
(**a**): Stationary distribution $f^{\mathrm{st}}\left(x_{T}\right)$ of total resources $x_{T}$, for four values of *N*, Nu=0.01, and q/λ=0.1. (**b**): Coefficient of variation $C_{V}$ as a function of *N*. The dashed curve is a B-spline approximation included as a guide to the eye. All results are estimations obtained from numerical solutions of Equation (8) along $2\times 10^{8}$ time steps.

Figure 4a presents numerical estimations of the stationary distribution of $x_{T}$ along time, in realizations of Equation (8) for different system sizes *N*. In all cases, $f^{\mathrm{st}}\left(x_{T}\right)$ is sharply peaked around a large value $x_{T}\approx 0.93$, and exhibits a broad shoulder for smaller $x_{T}$. Overall, this behavior is compatible with the analytically predicted value, $x_{T}\approx 0.901$, obtained from Equation (12). Note however the rather slow change of the shoulder at small $x_{T}$ as *N* grows: a variation by a factor of $10^{3}$ in the size of the population leads to a decrease of just above one order of magnitude in the height of the distribution in that zone.

This weak dependence on *N* is remarkably apparent in the coefficient of variation of $x_{T}$, defined as

(14) $$C_{V}=\frac{1}{\langle x_{T}\rangle }\sqrt{\frac{1}{T}\int_{0}^{T}{\left[x_{T}\left(t\right)-\langle x_{T}\rangle \right]}^{2}dt},$$

where

(15) $$\langle x_{T}\rangle =\frac{1}{T}\int_{0}^{T}x_{T}\left(t\right)dt$$

is the time average of $x_{T}\left(t\right)$, and *T* is a sufficiently long averaging interval. The coefficient $C_{V}$ encompasses overall statistical properties of $f^{\mathrm{st}}\left(x_{T}\right)$ in a single quantity, as a measure of the fluctuations of $x_{T}\left(t\right)$ relative to its average. Figure 4b is a log-log plot of $C_{V}$ as a function of *N*. Across the five orders of magnitude covered by the system sizes, the coefficient of variation only decreases by a factor of 3, and there is no clear indication that it might approach zero as N→∞. In fact, within this rather wide interval of *N*, it lacks the typical power-law trend that characterizes the system-size dependence of fluctuations in self-averaging statistical systems (usually, $N^{-z}$ with 0<z<1) [25]. This hints at a strongly heterogeneous behavior within the population, and calls for a closer look at the time evolution of individual replicators.

### 4.2. Heterogeneity and Clustering in the Evolution of Resources

**Figure 5:**
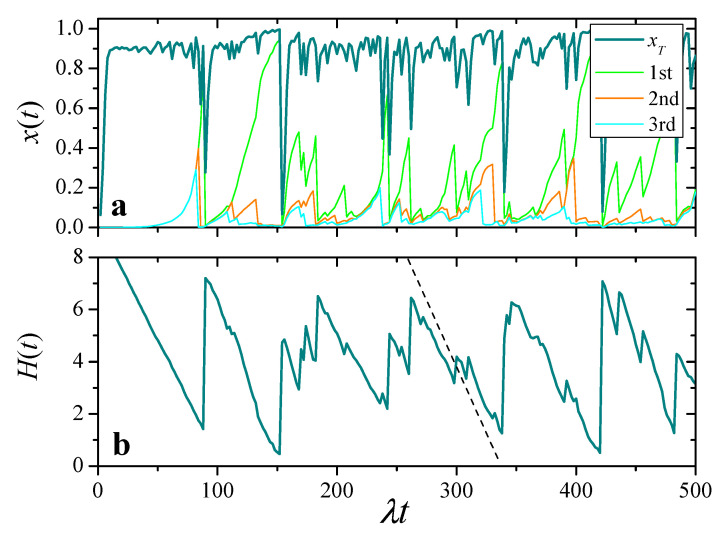
(**a**): Evolution of total resources, $x_{T}\left(t\right)$, and of individual resources for the three replicators with largest $x_{i}\left(t\right)$ at each time, in a realization with $N=10^{4}$, Nu=0.01, and q/λ=0.1. (**b**): Entropy of individual shares, Equation (16), for the same realization. The dashed segment has the slope analytically predicted for the decrease of H(t) with the two-cluster model of Section 4.3.

The darkest curve in Figure 5a shows the evolution of total resources $x_{T}\left(t\right)$ in a population of $N=10^{4}$ replicators, with Nu=0.01 and q/λ=0.1. At the initial time, all the replicators have identical resources, x(0)=u. We see that, most of the time, $x_{T}\left(t\right)$ fluctuates close to its maximum value. Intermittently, however, total resources exhibit sharp collapses where $x_{T}\left(t\right)$ suddenly drops to a small value, followed by a rapid recovery.

Other curves in Figure 5a show $x_{i}\left(t\right)$ for the three agents with highest resources at each time. These curves demonstrate the typically heterogeneous resource distribution over the population: most of the time, these three replicators accumulate a large fraction of the total resources. Comparison with $x_{T}\left(t\right)$, moreover, illustrates how collapses in total resources usually coincide with a reset event of the richest replicator.

As a more compact characterization of heterogeneity in the distribution of resources over the population, we have computed the entropy of the individual shares $x_{i}/x_{T}$ as a function of time:

(16) $$H\left(t\right)=-\sum_{i=1}^{N}\frac{x_{i}\left(t\right)}{x_{T}\left(t\right)}\ln\frac{x_{i}\left(t\right)}{x_{T}\left(t\right)}.$$

This quantity is depicted in Figure 5b for the same realization as in the upper panel. It shows that, in the intervals between collapses of $x_{T}\left(t\right)$, resources progressively accumulate in less and less replicators. Resetting of one of the replicators with high resources, in turn, entails a sudden growth of H(t), with an ensuing decrease as resources become increasingly concentrated.

During the intervals between collapses, we expect the population to be divided into at least two groups with different resource distributions inside each group. Those replicators that have undergone a reset event since the latest collapse should have low resources, close to the resetting level *u*. On the other hand, replicators that have evolved without resource resetting in the same period should possess, on the average, relatively higher resources, with a distribution closer to the equilibrium profile of Equation (10). In a succession of several consecutive collapses, the same mechanism may generate more than two groups, leading to a clustered, markedly heterogeneous resource distribution.

**Figure 6:**
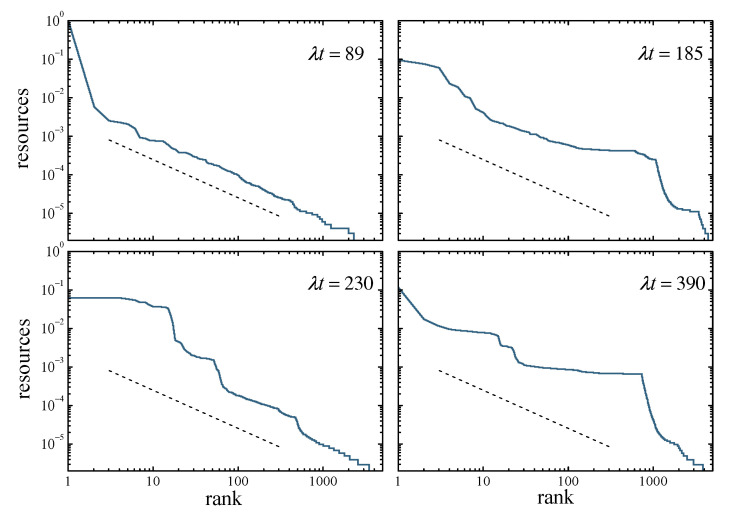
Four snapshots of a Zipf plot of individual resources versus rank in a decreasing list of resources, in a population of N=5000 replicators with Nu=0.01 and q/λ=0.1. The dashed segments show the slope that the plot should exhibit if the population had reached the equilibrium distribution of Equation (10). Plateaus of different sizes at different times reveal the formation of groups and, thus, clustering in the resource distribution.

Clustering in the resource distribution is well illustrated by a Zipf plot, in which individual resources are represented against the rank of each replicator in a list sorted by decreasing values of $x_{i}$. Figure 6 shows snapshots of this kind of plot at four times, in a system of N=5000 replicators. Other parameters are as in Figure 5. For λt=89, the first collapse has not taken place yet. In this situation, except for the first-rank replicator which already monopolizes practically all resources, the distribution over the population closely follows the equilibrium profile, whose slope is shown by the dashed line. As time elapses, the occurrence of collapses creates clusters, which in the Zipf plots appear as more or less flat plateaus separated by much sharper steps. In the Supplementary Video S1, which shows an animation of the Zipf plots for the same realization along time, the appearance, evolution, and fading of these plateaus is apparent.

Intermittent collapses of total resources and the consequent clustering of resource distribution, leading to an overall highly non-uniform behavior inside the population, are likely determinants of the differences observed between analytical and numerical results, as illustrated by Figure 3, and the slow decay of fluctuations of Figure 4b. In the following, under a few simplifying assumptions, we provide a stylized description for the behavior of the entropy H(t) and a prediction for the typical time between collapses, as well as an argument which explains the extremely slow decay of fluctuations in total resources as the system size grows.

### 4.3. Two-Cluster Model and the Decay of Fluctuations

As a simplified analytical approach to heterogeneity in the replicator population, we propose a toy model in which, at all times between collapses, total resources have the value $x_{T}$ given by Equation (12), and the ensemble is divided into just two clusters. The first cluster contains the $N_{r}\left(t\right)$ replicators whose resources have been reset after the latest collapse, occurred at time $t_{c}$. The second cluster comprises the $N-N_{r}\left(t\right)$ remaining replicators. Moreover, we assume that the individual resources in the first cluster are all equal to the reset level *u*, while the remaining resources are homogeneously distributed over the second cluster. This implies that the total resources in each cluster are $N_{r}\left(t\right)u$ and $x_{T}-N_{r}\left(t\right)u$, respectively. With these assumptions, Equation (16) yields

(17) $$H\left(t\right)=-\left[1-\frac{N_{r}\left(t\right)u}{x_{T}}\right]\ln\frac{1-\frac{N_{r}\left(t\right)u}{x_{T}}}{N-N_{r}\left(t\right)}-\frac{N_{r}\left(t\right)u}{x_{T}}\ln\frac{u}{x_{T}}\approx \ln\left[N-N_{r}\left(t\right)\right],$$

where the approximation of the rightmost side holds for $u\ll x_{T}$.

As successive reset events occur, replicators from the cluster of high resources are transferred to the other cluster at rate *q* so that, on the average, the number of replicators in the former satisfies the equation

(18) $$\frac{d}{dt}\left[N-N_{r}\left(t\right)\right]=-q\left[N-N_{r}\left(t\right)\right],$$

with $N-N_{r}\left(t_{c}\right)=N$ at the time of the latest collapse. Namely,

(19) $$N-N_{r}\left(t\right)=Ne^{-q(t-t_{c})}.$$

Replacing into the approximation for the entropy in Equation (17), we find

(20) $$H\left(t\right)\approx \ln N-q\left(t-t_{c}\right),$$

which predicts an approximate linear decay between collapses. The slanted dashed segment in Figure 5b has the slope predicted by this result, displaying very good agreement with the behavior of the numerically obtained signal for H(t).

Our approximation for the entropy H(t) makes it also possible to estimate the typical time between collapses, τ. In fact, in the two-cluster model a collapse will occur when just a single replicator remains in the high-resource cluster, $N-N_{r}\left(t\right)=1$, accumulating essentially all the resources. In this case, H=0 which, according to Equation (17), is the entropy attained at time $t=t_{c}+q^{-1}\ln N$. On the average, the last replicator will be reset after an additional time $q^{-1}$. Thus, we have

(21) $$\tau =\frac{1+\ln N}{q}.$$

**Figure 7:**
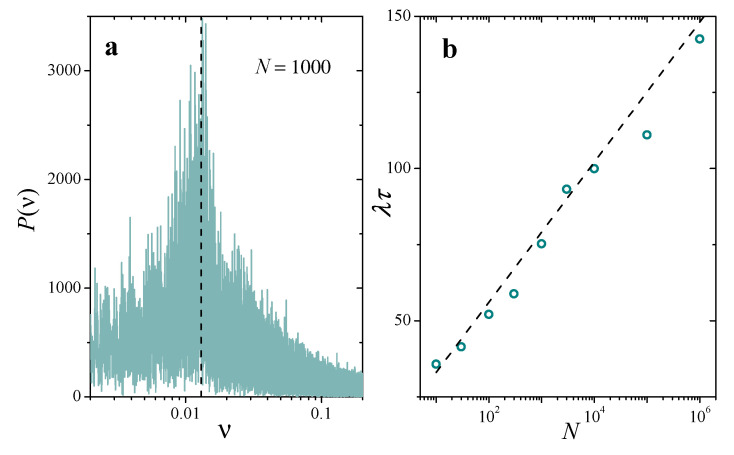
(**a**): Power spectrum of a time signal for the entropy H(t), numerically obtained in a replicator population with N=1000, Nu=0.01, and q/λ=0.1. The vertical dashed line is the frequency predicted for H(t) by the two-cluster model, in the approximation Nu≪1. (**b**): Average time between collapses estimated from the power spectrum of the entropy (symbols) and from the analytical prediction ((21), dashed line), as a function of *N*, with the same parameters as in panel (**a**).

In our simplified picture, τ is nothing but the period of the successive decays of H(t) between its maximum and its minimum. Figure 7a shows the power spectrum P(ν) of an actual numerical calculation of H(t) in a system with N=1000, Nu=0.01, and q/λ=0.1. Its broad profile exposes the stochastic nature of the mechanisms at play in the variation of the entropy, but shows a clear peak at a well-defined frequency, which reveals an underlying time-periodic pattern. The vertical dashed line demonstrates that this frequency coincides quite sharply with the prediction of Equation (21), $\nu =\tau^{-1}=q/\left(1+\ln N\right)$. We have performed this same comparison for different values of *N*, evaluating the main period of of numerical signals for the entropy from the position of the highest peak in their power spectra. In Figure 7b, results are compared with Equation (21), represented by the dotted line, with very good agreement.

Finally, along the same lines of approximation, we are able to give an explanation for the extremely slow decay of fluctuations in the total resources $x_{T}$ as the system size *N* grows, revealed by the weak dependence on *N* of the stationary resource distributions $f^{\mathrm{st}}\left(x\right)$ and $f^{\mathrm{st}}\left(x_{T}\right)$ (Figure 3 and Figure 4a) and explicitly illustrated in Figure 4b. The time signal of $x_{T}\left(t\right)$ shown in Figure 5a suggests that fluctuations in total resources are mainly dominated by the collapses associated with resetting of the replicators that accumulate most of the resources. In a highly stylized model for the signal $x_{T}\left(t\right)$, we can assume that the statistical distribution of total resources is given by a dichotomic process, where—in the interval between collapses—$x_{T}$ stays at its minimum value Nu during a “recovery time” $t_{R}$, and at its (approximate) equilibrium value 1−q/λ during the (average) remaining time $\tau -t_{R}$. Namely,

(22) $$f^{\mathrm{st}}\left(x_{T}\right)=\frac{t_{R}}{\tau }\delta \left(x_{T}-Nu\right)+\left(1-\frac{t_{R}}{\tau }\right)\delta \left(x_{T}-1+\frac{q}{\lambda }\right).$$

From this Ansatz, the calculation of the mean value and the standard deviation of $x_{T}$ is straightforward. In the limit Nu≪1, we find

(23) $$\langle x_{T}\rangle =\left(1-\frac{t_{R}}{\tau }\right)\left(1-\frac{q}{\lambda }\right),\;\;\;\;\;\sigma_{x_{T}}=\sqrt{\frac{t_{R}}{\tau }\left(1-\frac{t_{R}}{\tau }\right)}\left(1-\frac{q}{\lambda }\right),$$

which yields a coefficient of variation

(24) $$C_{V}=\sqrt{\frac{t_{R}/\tau }{1-t_{R}/\tau }}.$$

If $t_{R}$ is interpreted as the time needed by $x_{T}\left(t\right)$ to recover from its small value just after a collapse up to its equilibrium value, we do not expect $t_{R}$ to depend on *N*, at least for sufficiently large systems. Indeed, according to Equation (8), total resources should approximately obey ${\dot{x}}_{T}=\lambda x_{T}\left(1-x_{T}\right)-qx_{T},$ which is independent of *N*. If this is the case, Equations (21) and (24) imply that the coefficient of variation of $x_{T}$ decays as

(25) $$C_{V}∼\frac{1}{\sqrt{\ln N}}$$

for N→∞.

**Figure 8:**
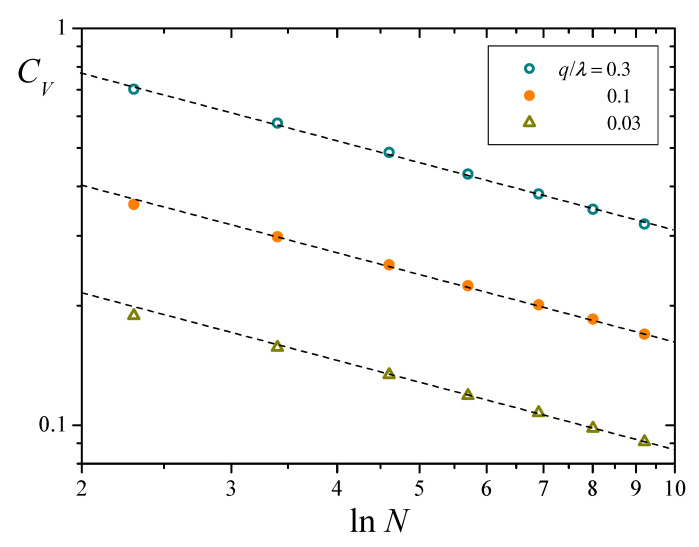
Coefficient of variation of total resources $C_{V}$ as a function of lnN, for Nu=0.01 and three values of q/λ (note log-log scales). Symbols correspond to numerical results, and dashed lines stand for the asymptotic behavior predicted in Equation (25).

Symbols in Figure 8 correspond to results for $C_{V}$ as a function of lnN for three different values of q/λ, obtained from numerical solutions of Equation (8) analogous to those of Figure 4b. Dashed lines stand for the asymptotic behavior predicted by Equation (25). Numerical results closely follow the prediction, even for relatively small values of *N*. On the one hand, Equation (25) shows that $C_{V}$ converges to zero as *N* grows, which validates the Chapman-Kolmogorov formulation for sufficiently large systems. On the other, the same result proves the extremely slow decay of fluctuations with the population size. Just as an illustration, suppose that one wants to diminish fluctuations in $x_{T}$ by a factor of 10, starting from results for a system of $10^{4}$ replicators. The new system should have nothing less than $10^{400}$ replicators (!), a size clearly beyond the reach of any presently available computational means.

## 5. Conclusions

Replicator dynamics with constant fitnesses is a basic model of agent competition, where one or a few agents eventually accumulate all the available resources. In this paper, we have investigated whether this concentration can be mitigated by stochastic resetting in the case of a homogeneous population. Reset events are randomly distributed in time, and force the dynamics of randomly drawn agents to start anew from a small value. Analytical results based on the Chapman-Kolmogorov equation show that, in fact, the long-time distribution of individual resources approaches a smooth profile, with a power-law decay of probability as the amount of resources grows.

However, numerical evidence reveals that—even for long times and large populations—the analytical prediction is, at most, an approximation to the actually observed resource distributions. A closer inspection of the dynamics of individual agents shows that the overall behavior is still governed by a few agents, which occasionally accumulate most of the total resources. When the resources of one of these wealthier agents are reset, total resources “collapse”, and the resource distribution suddenly becomes much more even. Subsequent collapses of this kind lead the distribution to develop clustering, separating the population into groups of agents with similar individual resources. This heterogeneity is responsible for the sustained differences between numerical and analytical results. These collapse-driven dynamics are also responsible for the extremely slow decay of fluctuations with the system size, which jeopardizes the use of the mean-field approach implicit in the Chapman-Kolmogorov Equation (6) for any practically attainable number of agents. Such anomalous statistical behavior is reminiscent of extreme-value statistics, whose relevance to economic processes has been emphasized in various contexts [20,26,27].

The present study complements recent work on cooperative agents subject to stochastic resetting [19], where we have shown that cooperation leads to resource redistribution, distorting the power-law distributions derived from the sole effect of reset events. These contributions represent a first attempt to characterize the collective behavior of interacting agents under the action of resetting, thus combining deterministic dynamics with stochastic ingredients. Other interactions of economic and ecological interest (e.g., parasitism, predator-prey, etc.) are worth considering in future work on the subject.

## Data Availability

Not applicable.

## Supplementary Materials

The following supporting information can be downloaded at: https://www.mdpi.com/article/10.3390/e25010099/s1, Video S1: Animation of Zipf plots for resource distribution over the replicator population (cf. Figure 6).

## References

[1] Dawkins R. (1978). Replicator selection and the extended phenotype 3. Z. Tierpsychol.

[2] Dawkins R. (1982). The Extended Phenotype.

[3] Ball J.A. (1984). Memes as replicators. Ethol. Sociobiol..

[4] Maynard Smith J. (1982). Evolution and the Theory of Games.

[5] Schuster P., Sigmund K. (1983). Replicator dynamics. J. Theor. Biol..

[6] Hofbauer J., Sigmund K. (1998). Evolutionary Games and Population Dynamics.

[7] Eigen M. (1971). Selforganization of matter and the evolution of biological macromolecules. Naturwissenschaften.

[8] Evans M.R., Majumdar S.N., Schehr G. (2020). Stochastic resetting and applications. J. Phys. A Math. Theor..

[9] Zanette D.H., Manrubia S.C. (1997). Role of intermittency in urban development: A model of large-scale city formation. Phys. Rev. Lett..

[10] Manrubia S.C., Zanette D.H. (1999). Stochastic multiplicative processes with reset events. Phys. Rev. E.

[11] Zanette D.H., Manrubia S.C. (2020). Fat tails and black swans: Exact results for multiplicative processes with resets. Chaos.

[12] Newman M.E.J. (2004). Power laws, Pareto distributions and Zipf’s law. Contemp. Phys..

[13] Burlando B. (1990). The fractal dimension of taxonomic systems. J. Theor. Biol..

[14] Gabaix X. (2009). Power laws in economics and finance. Ann. Rev. Econ..

[15] Riascos A.P., Boyer D., Herringer P., Mateos J.L. (2020). Random walks on networks with stochastic resetting. Phys. Rev. E.

[16] Daly E., Porporato A. (2010). Effect of different jump distributions on the dynamics of jump processes. Phys. Rev. E.

[17] Roldán E., Lisica A., Sánchez-Taltavull D., Grill S.W. (2016). Stochastic resetting in backtrack recovery by RNA polymerases. Phys. Rev. E.

[18] Abdoli I., Sharma A. (2021). Stochastic resetting of active Brownian particles with Lorentz force. Soft Matter.

[19] Gómez Garay I.T., Zanette D.H. (2021). Collective behavior of coupled multiplicative processes with stochastic resetting. J. Phys. Complex..

[20] Taleb N.N. (2005). Fooled by Randomness. The Hidden Role of Chance in Life and in the Markets.

[21] Taleb N.N. (2007). The Black Swan: The Impact of the Highly Improbable.

[22] Gardiner C.W. (1997). Handbook of Stochastic Methods.

[23] Kloeden P.E., Platen E. (1999). Numerical Solution of Stochastic Differential Equations.

[24] Drazin P.G. (1992). Nonlinear Systems.

[25] Lifshitz I. (2011). Self-Averaging.

[26] Embrechts P., Klüppelberg C., Mikosch T. (1997). Modelling Extremal Events: For Insurance and Finance.

[27] Novak S.Y. (2011). Extreme Value Methods with Applications to Finance.

