# Supplementary figures and images for: Resource Concentration and Clustering in Replicator Dynamics with Stochastic Reset Events
Source: Entropy (Basel). 2023 Jan 3;25(1):99. doi: 10.3390/e25010099 (PMC9858174; doi:10.3390/e25010099)

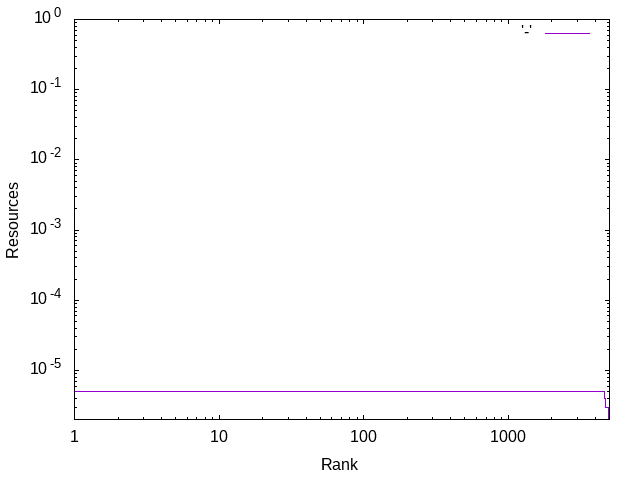

Supplement: Supplementary file 1 [file entropy-25-00099-s001.zip › entropy-2057052-supplementary.gif]
